# Overlooked Risk for Chronic Kidney Disease after Leptospiral Infection: A Population-Based Survey and Epidemiological Cohort Evidence

**DOI:** 10.1371/journal.pntd.0004105

**Published:** 2015-10-09

**Authors:** Huang-Yu Yang, Cheng-Chieh Hung, Su-Hsun Liu, Yi-Gen Guo, Yung-Chang Chen, Yi-Ching Ko, Chiung-Tseng Huang, Li-Fang Chou, Ya-Chung Tian, Ming-Yang Chang, Hsiang-Hao Hsu, Ming-Yen Lin, Shang-Jyh Hwang, Chih-Wei Yang

**Affiliations:** 1 Kidney Research Center, Department of Nephrology, Chang Gung Memorial Hospital, Chang Gung University, College of Medicine, Taoyuan, Taiwan; 2 Bloomberg School of Public Health, Johns Hopkins University, Baltimore, Maryland, United States of America; 3 Department of Family Medicine, Chang Gung Memorial Hospital, Chang Gung University College of Medicine, Taoyuan, Taiwan; 4 Division of Nephrology, Department of Internal Medicine, Kaohsiung Medical University Hospital, Kaohsiung, Taiwan; University of California San Diego School of Medicine, UNITED STATES

## Abstract

**Background:**

Leptospirosis is the most widespread zoonosis. Chronic human infection and asymptomatic colonization have been reported. However, renal involvement in those with leptospira chronic exposure remains undetermined.

**Methods and Findings:**

In 2007, a multistage sampling survey for chronic kidney disease (CKD) was conducted in a southern county of Taiwan, an area with a high prevalence of dialysis. Additionally, an independent cohort of 88 participants from a leptospira-endemic town was followed for two years after a flooding in 2009. Risks of CKD, stages of CKD, associated risk factors as well as kidney injury markers were compared among adults with anti-leptospira antibody as defined by titers of microscopic agglutination test (MAT). Of 3045 survey participants, the individuals with previous leptospira exposure disclosed a lower level of eGFR (98.3±0.4 vs 100.8±0.6 ml/min per 1.73 m^2^, *P*<0.001) and a higher percentage of CKD, particularly at stage 3a-5 (14.4% vs 8.5%), than those without leptospira exposure. Multivariable linear regression analyses indicated the association of leptospiral infection and lower eGFR (95% CI -4.15 to -1.93, *P* < 0.001). In a leptospiral endemic town, subjects with a MAT titer ≥400 showed a decreased eGFR and higher urinary kidney injury molecule–1 creatinine ratio (KIM1/Cr) level as compared with those having lower titers of MAT (P<0.05). Furthermore, two participants with persistently high MAT titers had positive urine leptospira DNA and deteriorating renal function.

**Conclusions and Significance:**

Our data are the first to show that chronic human exposure of leptospirosis is associated significantly with prevalence and severity of CKD and may lead to deterioration of renal function. This study also shed light on the search of underlying factors in areas experiencing CKD of unknown aetiology (CKDu) such as Mesoamerican Nephropathy.

## Author Summary

Chronic kidney disease (CKD) has a high and increasing worldwide prevalence.

Leptospirosis, an important re-emerging infectious disease caused by the pathogenic spirochete *Leptospira*, is the most widespread zoonosis throughout the world, particularly in tropical and subtropical regions. Chronic human infection and asymptomatic colonization have been reported. However, the evidence of renal involvement in those with leptospira exposure history or human carrier remains undetermined. In this study we found that those individuals with previous leptospira exposure disclosed a lower renal function and a higher percentage of CKD. Additionally, in our cohort study, those with a high serum titer by leptospira agglutination test showed decreased renal function and higher kidney injury marker. We are the first to identify the association between CKD and leptospiral infection. This information may provide a novel approach for CKD of unknown aetiology but also significantly impact global control of leptospirosis and CKD burden.

## Introduction

Chronic kidney disease (CKD) has an increasing prevalence worldwide [1]. According to the recent report on the global burden of diseases, there were 16.3 CKD-associated deaths per 100 000 individuals in 2010, ranking 18^th^ in the list of 86 causes. Compared to its ranking of 27^th^ in 1990, the rate of increasing has been only second to HIV and AIDS [2]. To reduce the global burden of CKD, effective strategies for the detection and prevention of CKD are needed. Diabetes and hypertension are the leading causes of CKD in all developed countries and in some developing countries [1,3]. In the developing world, besides environmental and occupational exposure to chemicals, infections are important causes of renal failure [3].

Leptospirosis, caused by the pathogenic spirochete *Leptospira*, is the most widespread zoonosis throughout the world, particularly in tropical and subtropical regions [4]. This is an important re-emerging infectious disease with a huge public health impact because of its increasing incidence and epidemic proportions [5]. Almost every mammal, but mainly rodents, can serve as a carrier of leptospira in the proximal renal tubules of the kidneys, from which leptospira are shed into the urine [4,6]. The pathogen is transmitted to humans through contaminated soil, water, or infected animal urine. Multiple organ involvement [7] may be encountered in acute severe leptospirosis, and renal involvement is commonly characterized by tubulo-interstitial nephritis and tubular dysfunction [8–10]. Besides protean acute presentations of leptospirosis, the possibility of chronic human infection was suggested with only sporadic cases [11], such as late onset uveitis with leptospiral DNA amplified from aqueous humor [12], central sleep apnea due to chronic neuroleptospirosis [13] and a recent report of neuroleptospirosis diagnosed by next-generation sequencing [14]. Recently, asymptomatic human renal colonization of leptospira in an area of high disease transmission was reported [15]. In the Central American nations where leptospirosis is endemic, leptospirosis was speculated as one of the possible causes of Mesoamerican nephropathy, a form of chronic kidney disease with unknown origin prevailed in young male agricultural workers [16]. However, the pathogenic significance, such as chronic kidney damage, is uncertain. In the current study, we performed a community-based survey to evaluate the risk of CKD in participants with leptospiral infection. Moreover, a two-year cohort study was conducted in the region with high transmission rates of leptospiral diseases after a natural flood. We postulated that subjects with leptospiral infection, as defined by positive microscopic agglutination test (MAT), possessed an increased risk of CKD compared with those without leptospiral infection.

## Methods

### Ethics statement

This study complied with the guidelines of the Declaration of Helsinki and was approved by the Medical Ethics Committee of Chang Gung Memorial Hospital, a tertiary referral center located in the northern part of Taiwan. Approval from the Institutional Review Board was obtained with specific written informed consent from patients. For any participants under the age of 18, a parent or guardian provided informed consent. Furthermore, not only were all data securely protected (by delinking identifying information from the main data sets) and made available only to investigators, but they were also analyzed anonymously. Finally, all primary data were collected according to procedures outlined in epidemiology guidelines that strengthen the reporting of observational studies.

### Study design and study setting

In 2007, a single-time active surveillance for leptospirosis was performed on residents older than age 15 years (n = 1,026,288) in a Southern County of Taiwan. A multistage, stratified sampling method was used for selecting 3000 participants from the Household Register Database. In the first stage, in addition to the major city comprising 27.3% of the county population, 8 of the 27 county villages were randomly selected. In the next stage, a total of 50 districts (Li; the smallest unit of administration) from the city and the eight chosen villages were selected based on the sampling probability proportional to the population size of the city or the selected village. In the final stage, 60 participants were randomly selected from each of the 50 selected districts. Assuming a response rate of approximately one sixth, 18,000 participants were selected by the abovementioned approach and invited for the CKD screening program.

In August 2009, Typhoon Morakot flooding led to a biggest local outbreak of leptospirosis in the leptospira-endemic town within the Southern County in recent years [17,18]. To evaluate the long-term health effect of leptospiral infection, a baseline survey was conducted on a cohort of the town residents three months after the typhoon in 2009. All cohort members were invited again for a follow-up visit in 2011. No known acute leptospirosis record was reported in these residents.

### Measurement of predictors and covariates

Blood, urine, and biochemical tests were performed on the study participants at enrolment. The MAT, a gold standard test for serological investigation and diagnosis in leptospirosis, was applied to sera of all participants, with most prevalent serovar. As *Leptospira santarosai* serovar Shermani is the major and most prevalent servoar in Taiwan and up to 72.2–77.3% of acute infection was related to this serovar [8,19], MAT for this serovar was performed for analysis and expressed as anti-leptospira antibody seropositivity. MAT titers were reported as the reciprocal of the number of dilutions still with 50% of live bacterial antigen agglutination [20]. Cases were defined as sero-positive given a MAT titer ≥ 1: 100, indicative of past exposure [4].

### Measurement of outcomes

Outcomes were renal function, CKD and stages of CKD. CKD was diagnosed according to the KDIGO 2012 Clinical Practice Guidelines and classified based on eGFR (estimated glomerular filtration rate) category and albuminuria category [21]. Microalbuminuria was defined as a urinary albumin-to-creatinine ratio of 30 mg/g or higher using the first-morning urine. The eGFR was calculated using the Chronic Kidney Disease Epidemiology Collaboration (CKD-EPI) equation, which is more accurate than the Modification of Diet in Renal Disease Study equation [22].

In the prospective cohort study, deterioration of renal function and kidney injury biomarkers are two additional outcomes. Four different biomarkers were applied in 2011, including serum and urine neutrophil gelatinase-associated lipocalin (NGAL), kidney injury molecule–1 creatinine ratio (KIM–1/Cr), and monocyte chemoattractant protein–1 (MCP–1). Urine DNA was extracted from the Cohort participants and tested for leptospira DNA detection using PCR primer sets as previously described [23,24].

### Statistical analysis

For the K-County study population, participant characteristics were described overall and by the prevalence of a positive leptospiral serum testing result (as defined by MAT titer ≥ 1:100). To reflect the multi-stage sampling scheme for the K-County study population, survey-based population estimates of participant characteristics were weighted by sampling probabilities [25]. Continuous variables were presented as mean ± SD and compared between groups of subjects by t-test or ANOVA whenever appropriate. Categorical factors were displayed in weighted percentages, and the Pearson chi-squared test or Fisher’s Exact test was used to compare these covariates among groups. A survey-based linear regression on the continuous eGFR score was used in the univariate and multivariate models to appropriately account for the multistage, stratified probability sampling procedure. Regardless of the statistical significance in the univariate models, all of the variables were retained in the multivariable models to adjust for potential confounding. Data were further stratified into two groups: DM (diabetes mellitus) and NDM (non-diabetes mellitus), and multivariate analyses were performed separately in each group.

For the W-Township cohort, demographics and clinical characteristics were compared among subjects who exhibited negative, low (MAT titer 1:100–200) or high (MAT titer ≥ 1:400) titer testing results at baseline. The eGFR scores of the same cohort were compared between 2009 and 2011, which was further presented among negative, low, and high MAT titers. The difference in the pre- and post- eGFR associated with MAT titer change was also evaluated.

All statistical analyses were performed using Stata software, version 12.0 (Stata Corp., College Station, TX). A two-sided significance level at 0.05 was considered statistically significant.

## Results

### Southern county survey population

The community-based, cross-sectional survey included 3,045 participants from the chosen southern county of Taiwan in 2007, with a response rate of approximately 16.9%. The mean age of the sample was 47.5 years and the mean age of the sampling pool population was 46.6 years.

Table 1 shows weighted results of the demographics, clinical characteristics and biomedical tests of the overall study population and by the MAT testing results. The anti-leptospira antibody-positive cases were older than anti-leptospira antibody-negative ones (47.8 vs. 45.0 years, P-value<0.01). The anti-leptospira antibody positive patient population also had a 1%-higher prevalence of diabetes mellitus (5.7% vs. 4.6%, P-value<0.01). However, the anti-leptospira antibody negative patient population had a higher prevalence of liver disease (5.2% vs. 7.6%, P-value<0.01) associated with higher positive rate of HBsAg (12.1% vs. 13.6%, P-value 0.012) and anti-HCV Ab (3.9% vs. 5.4%, P-value 0.02). Patients with positive anti-leptospira antibody disclosed a 2% lower GFR estimated by CKD-EPI (98.3 ml/min/1.73m^2^ vs. 100.8 ml/min/1.73m^2^, P-value<0.01).

**Table 1:** Characteristics of the southern county of Taiwan population overall and results of anti-leptospira antibody by microscopic agglutination test (MAT).

Characteristic		Anti-leptospira antibody		*P* Value^2^
	Overall (N = 3,045)	Positive (N = 1,034)	Negative (N = 2,011)	
**Demographic characteristic**	% (n/N)^1^	% (n/N)^1^	% (n/N)^1^	
**Age (y), mean± SD**	46.6 ± 0.7	47.8 ± 0.5	45.0 ± 0.9	<0.001^3^
**Male (%)**	48.8 (1484/3045)	48.5 (502/1034)	49.3 (982/2011)	0.6
**Years of education (%)**				
**6**	23.0 (802/3017)	23.3 (289/1024)	22.8 (513/1993)	1.0
**6–12**	48.5 (1399/3017)	48.0 (462/1024)	48.9 (937/1993)	
**≥13**	28.5 (816/3017)	28.7 (273/1024)	28.3 (543/1993)	
**Annual income (%)**				
**<13,000 (US dollars)**	52.1 (1612/2902)	49.0 (534/982)	53.5 (1078/1920)	0.15
**≥13,000 (US dollars)**	47.9 (1290/2902)	51.0 (448/982)	46.5 (842/1920)	
**Medical history (%)**				
**DM**	5.0 (172/3026)	5.7 (67/1025)	4.6 (105/2001)	<0.001
**Hypertension**	14.0 (456/3026)	14.0 (159/1025)	14.0 (297/2001)	1.0
**Cardiovascular disease**	4.8 (154/3026)	4.6 (46/1025)	5.0 (108/2001)	0.6
**Gout or hyperuricemia**	5.0 (154/3025)	5.1 (47/1025)	4.9 (107/2000)	0.8
**Urinary tract disease**	4.3 (135/3026)	4.8 (45/1025)	4.2 (90/2001)	0.2
**Health-related behaviors (%)**				
**Chinese herb use**	18.8 (564/3003)	19.6 (197/1019)	18.2 (367/1984)	0.1
**Oral analgesic use**	11.2 (321/2998)	11.5 (112/1015)	11.0 (209/1983)	0.8
**Analgesic injection**	5.2 (160/2983)	5.1 (55/1011)	5.4 (105/1972)	0.8
**Cigarette smoking**	25.2 (761/3020)	25.7 (269/1021)	24.7 (492/1999)	0.4
**Alcohol**	16.0 (502/3012)	15.2 (172/1019)	16.4 (330/1993)	0.2
**Metabolic syndrome**	18.3 (568/3045)	19.0 (202/1034)	18.0 (366/2011)	0.6
**Laboratory data**				
**Hemoglobin (g/dl), mean± SD**	14.2 ± 0.05	14.2 ± 0.08	14.2 ± 0.03	1.0^3^
**Positive for HBs Ag (%)**	12.9 (383/3045)	12.1 (123/1034)	13.6 (260/2011)	0.01
**Positive for anti-HCV Ab (%)**	5.0 (166/3045)	3.9 (45/1034)	5.4 (121/2011)	0.02
**eGFR (ml/min/1.73 m** ^**2**^ **), mean± SD**	100.0 ± 0.4	98.3 ± 0.4	100.8 ± 0.6	<0.001^3^

Abbreviations: eGFR, estimated GFR as calculated using the Chronic Kidney Disease Epidemiology Collaboration (CKD-EPI) equation; SD, standard deviation; DM, diabetes mellitus; HBs Ag, hepatitis B virus surface antigen; HCV, hepatitis C virus.

^1^Survey-based weighted percentage (raw n/N)

^2^P-values for Survey-based Chi-squared test

^3^P-values for survey-based t-test

**Fig 1:**
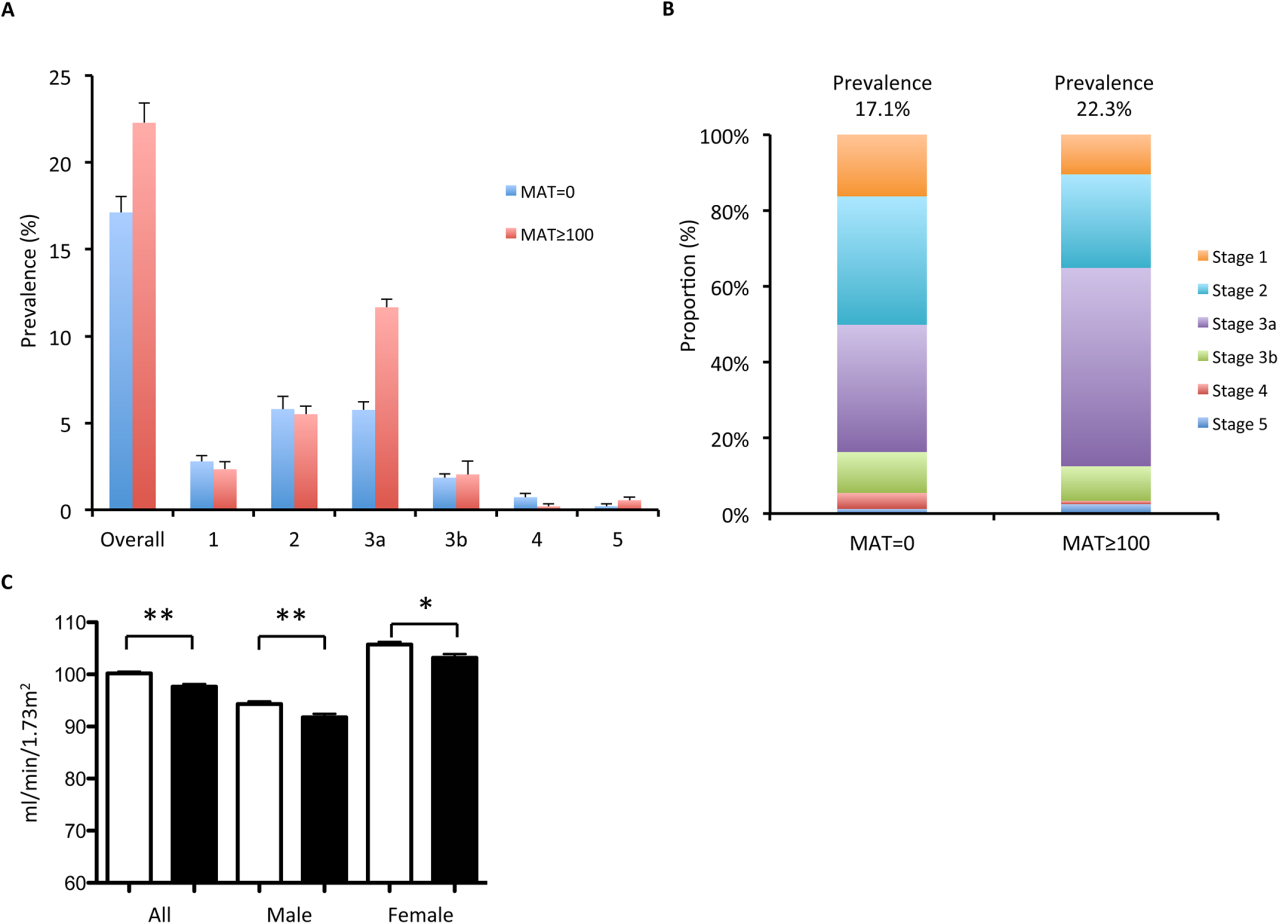
Prevalence and proportion of CKD (chronic kidney disease) stages by MAT titer zero and MAT titer ≥ 100 (A) Prevalence of CKD stages in MAT = 0 and MAT ≥ 100. (B) Proportion of CKD stages by in MAT = 0 and MAT ≥ 100. (C) eGFR (estimated glomerular filtration rate) in MAT = 0 (white bar) and MAT≥ 100 (black bar) of different groups (all, male, and female). **p<0.001.

Fig 1 shows the prevalence and proportion of CKD stages in anti-leptospira antibody positive and negative groups. The prevalence of CKD is significantly higher in anti-leptospira antibody positive group. Those individuals with positive anti-leptospira antibody disclosed higher percentage of CKD than those with a negative anti-leptospira antibody, particularly at stage 3a-5 (14.4% vs 8.5%). Both male and female participants with positive anti-leptospira antibody have lower eGFR.

In addition to exposure to *Leptospira*, important characteristics strongly associated with lower eGFR was included in the variables such as diabetes mellitus, hypertension, cardiovascular disease, gout, urinary tract infection, metabolic syndrome, Chinese herbs, and analgesics. Of the 18 variables (Table 2), anti-leptospira antibody serology (serum positive or serum negative) is one of the 13 variables related to lower eGFR by univariate linear regression analyses. After adjusting for 17 variables, multiple linear regression analyses revealed that subjects, with a positive anti-leptospira antibody, scored 3 less points than those with a negative anti-leptospira antibody (-3.0, 95% CI = -4.2, -1.9; P-value <0.001). Table 3 revealed the independent factors for deterioration of eGFR in DM and NDM. Leptospiral infection, age, hypertension, and the use of Chinese herb were significantly associated with lower eGFR in both DM and NDM groups.

**Table 2:** Results of Univariate and Multivariate Linear Regression on eGFR derived by CKD-EPI equation.

Variables	Univariate			Multivariate		
	β coefficient (95% CI)		*P* Value	β coefficient (95% CI)		*P* Value
**Seropositive to *Leptospira***	-4.05	(-4.99, -3.11)	<0.001	-3.04	(-4.15, -1.93)	<0.001
**Age (y)**	-0.78	(-0.86, -0.69)	<0.001	-0.74	(-0.78, -0.71)	<0.001
**Male**	-3.06	(-5.67, -0.44)	0.02	-1.49	(-2.73, -0.25)	0.02
**Annual income ≥13,000 (US dollars)**	4.50	(1.55, 7.46)	0.004	-1.10	(-2.20, 0.00)	0.05
**Diabetes Mellitus**	-14.61	(-17.15, -12.06)	<0.001	-2.41	(-4.84, 0.02)	0.05
**Hypertension**	-17.42	(-20.52, -14.32)	<0.001	-3.56	(-5.23, -1.90)	<0.001
**Cardiovascular disease**	-16.64	(-19.56, -13.72)	<0.001	-4.00	(-6.55, -1.46)	0.002
**Gout or Hyperuricemia**	-12.83	(-14.74, -10.92)	<0.001	-5.93	(-8.41, -3.45)	<0.001
**Urinary tract disease**	-7.79	(-9.89, -5.69)	<0.001	0.88	(-1.72, 3.48)	0.5
**Chinese herb use**	-0.79	(-2.86, 1.27)	0.4	-0.60	(-1.96, 0.75)	0.4
**Oral analgesic use**	-0.92	(-3.60, 1.75)	0.5	2.07	(0.20, 3.94)	0.03
**Analgesic injection**	-6.87	(-9.84, -3.89)	<0.001	-1.37	(-3.93, 1.19)	0.3
**Cigarette smoking**	-4.68	(-6.03, -3.32)	<0.001	-1.30	(-2.76, 0.17)	0.08
**Alcohol**	-3.89	(-5.22, -2.56)	<0.001	0.79	(-0.79, 2.35)	0.3
**Metabolic syndrome**	-11.25	(-13.62, -8.88)	<0.001	-1.26	(-2.76, 0.24)	0.1

**Table 3:** Results of Multivariate Linear Regression on eGFR derived by CKD-EPI Equation in DM versus NDM participants.

	DM			NDM		
	β coefficient (95% CI)		*P* Value	β coefficient (95% CI)		*P Value*
**Seropositive to *Leptospira***	-7.53	(-13.18, -1.9)	0.009	-2.84	(-3.97, -1.70)	<0.001
**Age (y)**	-0.85	(-1.09, -0.61)	<0.001	-0.75	(-0.79, -0.71)	<0.001
**Male**	2.46	(-4.91, 9.82)	0.5	-1.74	(-3.00, -0.48)	0.007
**Annual income ≥ 130,000 (US dollars)**	-2.69	(-9.32, 3.94)	0.4	-1.18	(-2.29, -0.06)	0.04
**Hypertension**	-10.00	(-16.28, -3.72)	0.002	-2.67	(-4.43, -0.92)	0.003
**Cardiovascular disease**	-7.80	(-16.94, 1.34)	0.09	-3.79	(-6.48, -1.10)	0.006
**Gout or Hyperuricemia**	-2.81	(-15.54, 9.93)	0.7	-6.15	(-8.67, -3.63)	<0.001
**Urinary tract disease**	-2.10	(-13.68, 9.49)	0.7	0.75	(-1.92, 3.42)	0.6
**Chinese herb use**	12.42	(5.27, 19.57)	0.001	-1.39	(-2.78, -0.001)	0.05
**Oral analgesic use**	6.78	(-5.67, 19.24)	0.3	1.84	(-0.05, 3.73)	0.06
**Analgesic injection**	-15.40	(-29.02, -1.77)	0.03	-0.63	(-3.26, 2.00)	0.6
**Cigarette smoking**	-0.21	(-7.33, 6.91)	1.0	-1.38	(-2.86, 0.11)	0.07
**Alcohol**	-3.77	(-11.67, 4.13)	0.3	0.93	(-0.67, 2.53)	0.3
**Metabolic syndrome**	-3.22	(-9.79, 3.35)	0.3	-0.74	(-2.29, 0.81)	0.4

DM: diabetes mellitus; NDM: Non-diabetes mellitus

### Endemic township two-year cohort

After typhoon Morakot, we obtained data for 101 participants who had experienced the typhoon flooding without obvious clinical symptoms in one township at the southern county in 2009. As of 2011, 88 participants had been followed-up since 2009, 86.4% of whom were positive for anti-leptospira antibody at baseline. Among these 88 subjects, 12 were negative for anti-leptospira antibody (mean age 61.7 years), 41 having an MAT titer between 100 and 200 (mean age 56.5 years), and 35 with MAT titer at least 400 (mean age 53.1 years, Table 4). The prevalence of diabetes mellitus, hypertension, micro-albuminuria did not show significant differences among three groups. Neither did subjects within three groups display significant differences in serum levels of creatinine, eGFR by CKD-EPI, or uric acid (Table 4).

**Table 4:** Characteristics of the cohort population overall and by grouped titers of anti-leptospira antibody by MAT.

Characteristic		Anti-leptospira antibody MAT titers				
	Total (n = 88)	MAT negative (n = 12)		MAT = 100–200 (n = 41)		MAT≥400 (n = 35)
**Age**	55.8 ± 15.0	61.7 ± 14.0		56.5 ± 14.1		53.1 ± 16.1
**Male (%)**	38.2	25.0		31.7		50.0
**BMI**	23.8 ± 3.3	22.3 ± 2.8		24.4 ± 3.5		23.6 ± 3.1
**Waist circumference (cm)**	81.6 ± 11.1	78.1 ± 9.6		82.7 ± 11.5		81.5 ± 11.2
**Hip circumference (cm)**	94.8 ± 7.7	92.7 ± 7.0		96.6 ± 8.1		93.5 ± 7.5
**Medical History**						
**Diabetes Mellitus (%)**	20.6	14.3		19.6		24.3
**Hypertension (%)**	26.3	14.3		36.4		18.9
**Microalbuminuria (%)**	14.4	14.3		15.2		13.5
**Laboratory data**						
**Creatinine (mg/dl)**	0.7 ± 0.2	0.7 ± 0.2		0.8 ± 0.2		0.8 ± 0.2
**eGFR (ml/min/1.73 m** ^**2**^ **)** ^**1**^	105.9 ± 17.4	104.6 ± 20.0		105.0 ± 16.3		107.4 ± 18.0
**Hematocrit (%), mean**± **SD**	41.7 ± 3.9	41.0 ± 4.1		41.2 ± 4.5		42.7 ± 2.7
**RBC (10** ^**6**^ **/**±**l)**	4.7 ± 0.6	4.4 ± 0.5		4.7 ± 0.5		4.8 ± 0.6
**Hemoglobin (g/dl)**	14.0 ± 1.6	13.8 ± 1.6		13.7 ± 1.9		14.3 ± 1.1
**BUN (mg/dl)**	13.6 ± 4.9	14.0 ± 2.9		14.1 ± 6.3		12.8 ± 3.1
**AST (IU/L)**	27.8 ± 10.9	26.5 ± 4.7		28.4 ± 13.2		27.4 ± 9.5
**ALT (IU/L)**	25.4 ± 12.8	23.6 ± 7.9		25.4 ± 14.4		26.4 ± 11.9
**HDL cholesterol (mg/dl)**	51.2 ± 13.3	49.9 ± 8.9		51.0 ± 12.8		51.8 ± 15.1
**LDL cholesterol (mg/dl)**	123.2 ± 28.8	130.3 ± 33.6		119.4 ± 25.9		125.3 ± 30.5
**Total Cholesterol (mg/dl)**	200.9 ± 32.2	208.0 ± 32.5		196.6 ± 33.2		203.4 ± 31.2
**Triglyceride (mg/dl)**	120.9 ± 107.6	92.6 ± 61.1		114.9 ± 60.9		137.4 ± 152.0
**Uric Acid (mg/dl)**	6.3 ± 1.6	5.5 ± 0.8		6.6 ± 1.8		6.2 ± 1.5

Values expressed with a plus/minus sign are the mean ± SD; AST indicates aspartate aminotransferase; ALT indicates alanine aminotransferase;

^1^The eGFR, estimated GFR, was calculated using the Chronic Kidney Disease Epidemiology Collaboration (CKD-EPI) equation

**Fig 2:**
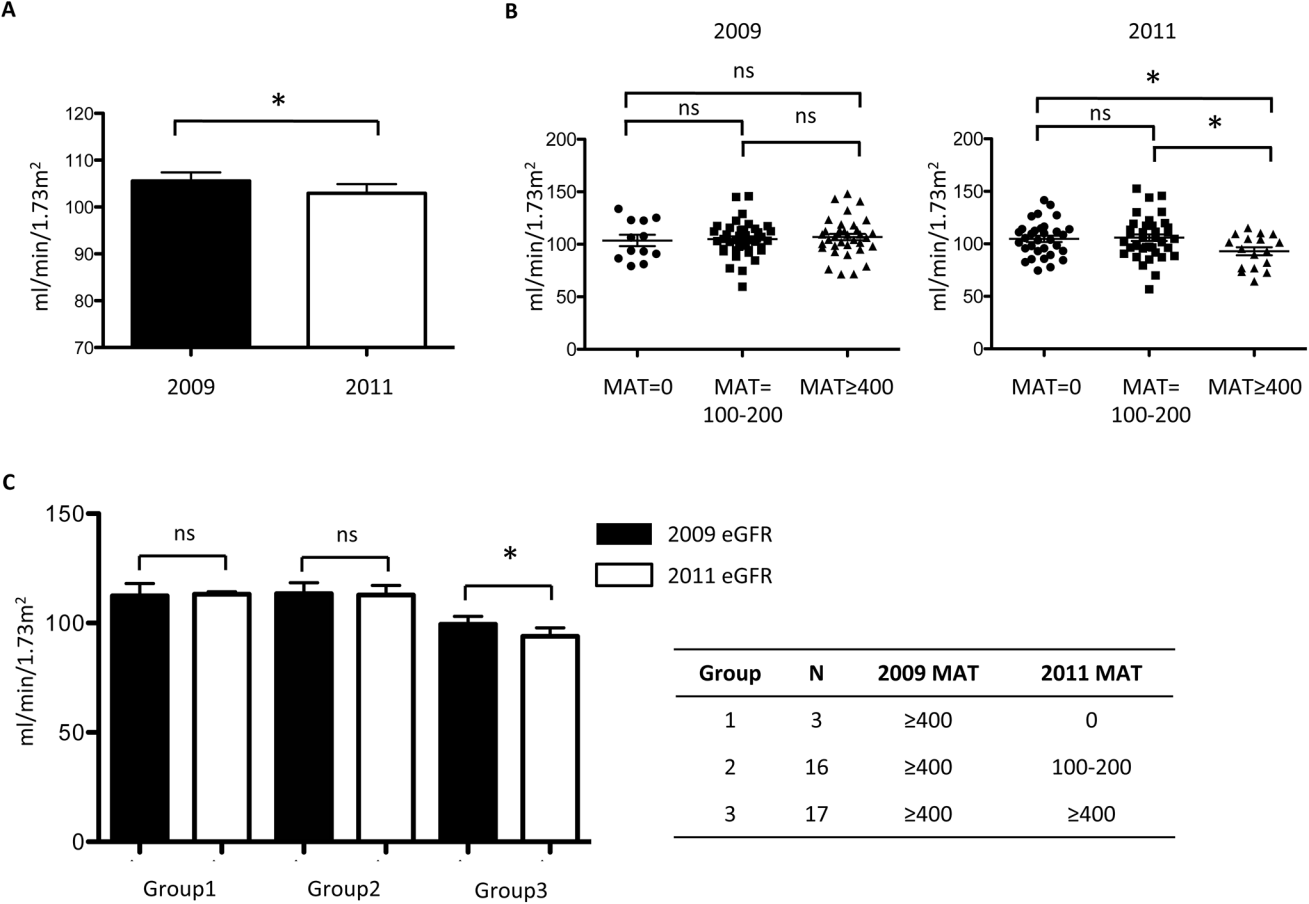
The eGFR in the township cohort study (n = 88). (A) eGFR for cohort in 2009 and 2011. (B) eGFR of participants grouped by three different MAT titers in two-year cohort of W township. (C) eGFR of participants with MAT≥400 in 2009 grouped by three different MAT titers change in two-year cohort of W township over time. * *p*<0.05.

eGFR by CKD-EPI of every participant was followed in 2011 (Fig 2). All these cases have significantly lower eGFR in 2011 (102.9 ± 18.3 ml/min/1.73m^2^) than in 2009 (105.7 ± 17.4 ml/min/1.73m^2^). In subgroup analysis, eGFR by CKD-EPI did not show a significant difference among three groups in 2009. However, in 2011, cases with MAT titer above 400 had, on average, a significantly lower eGFR (92.9 ± 15.8 ml/min/1.73m^2^) than that for participants with an MAT titer of 100–200 (105.9 ± 19.5 ml/min/1.73m^2^) and those with a negative MAT (104.7 ± 16.7 ml/min/1.73m^2^, P-value = 0.039). Since cases with MAT titer above 400 in 2011 all derived from cases with MAT titer above 400 in 2009, we further divided cases into three different groups: group 1: MAT≥400 in 2009 and MAT = 0 in 2011; group 2: MAT≥400 in 2009 and MAT = 100–200 in 2011; group 3: MAT≥400 in 2009 and MAT≥400 in 2011. Cases possess MAT titer above 400 in both time points disclosed deterioration of renal function in two-year follow up.

**Fig 3:**
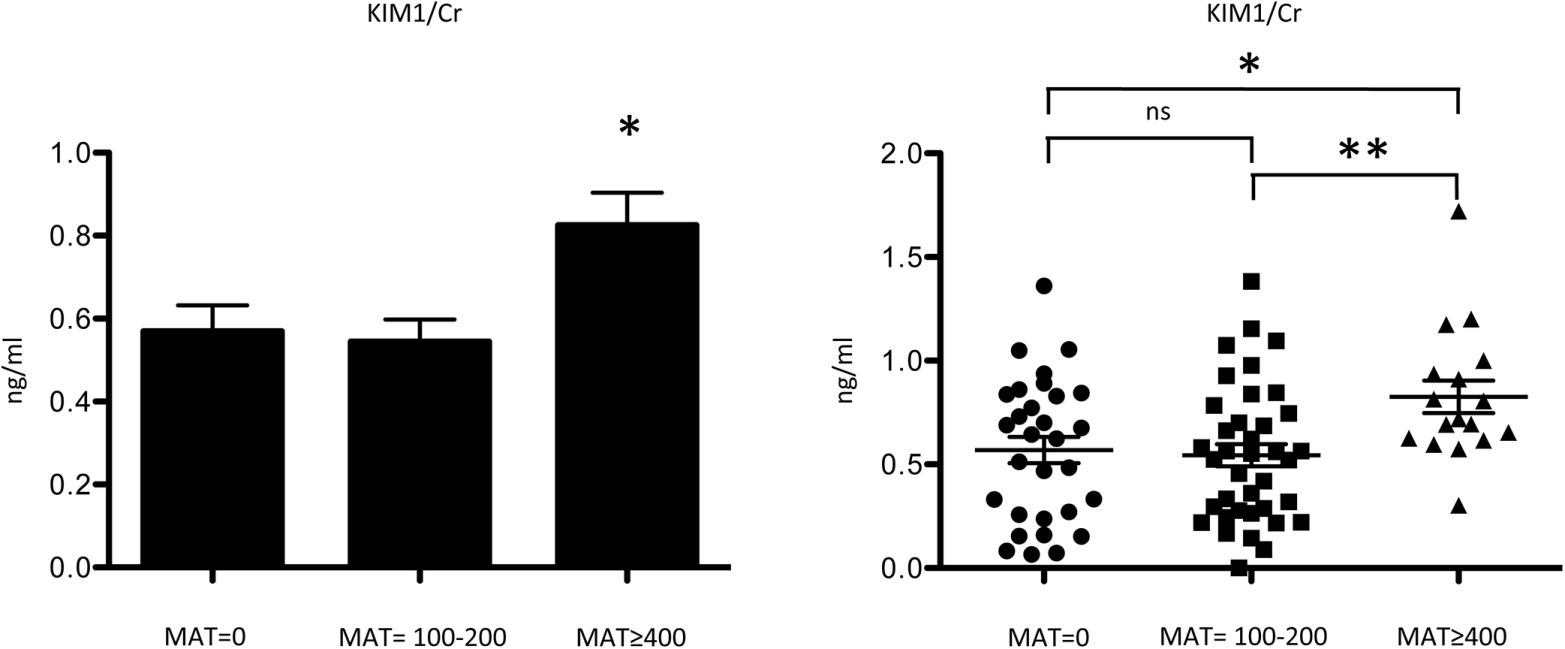
Kidney injury molecule–1 creatinine ratio (KIM1/Cr) level of three different MAT titer groups for township cohort in 2011 (Negative, titer = 100–200, titer ≥400). *p<0.05, **p<0.001.

To evaluate kidney injury, a set of biomarkers was tested in the 88 residents from W township in 2011. Cases also were divided into three groups according to MAT titer level (MAT = 0, MAT = 100–200, and MAT≥400). Urinary KIM1/Cr ratio is higher in cases with MAT titer above 400 as compared with that in the other two groups (0.6 ± 0.3 ng/mg vs. 0.5 ± 0.3 ng/mg vs. 0.8 ± 0.3 ng/mg, P-value<0.05) (Fig 3). The level of urinary, serum NGAL and serum MCP1 did not show a significant difference among three groups (Table 5).

**Table 5:** Comparison results of selected biomarkers associated with acute or chronic kidney injuries in the cohort population overall and by MAT titers at follow-up in 2011.

Biomarkers	Total (n = 88)	MAT = 0 (n = 32)	MAT = 100–200 (n = 39)	MAT≥400 (n = 17)
**NGAL Serum (ng/ml)**	78.5 ± 36.4	79.1 ± 39.9	74.8 ± 36.1	82.2 ± 34.0
**NGAL Urine (ng/ml)**	34.9 ± 63.2	45.4 ± 87.0	27.0 ± 47.2	31.0 ± 43.4
**KIM1/Cr (ng/mg)** ^**1**^ ^,^ ^**3**^ ^,^ ^**4**^	0.609 ± 0.346	0.569 ± 0.34	0.544 ± 0.327	0.826 ± 0.322
**MCP1 (pg/ml)**	229.5 ± 152.7	261.9 ± 177.3	194.8 ± 127.5	250.1 ± 148.6

^1^ *P*<0.05 for oneway ANOVA between MAT = 1, MAT≥ 100, and MAT≥400.

^2^ *P*<0.05 for MAT = 0 vs MAT≥ 100.

^3^ *P*<0.05 for MAT = 0 vs MAT≥ 400.

^4^ *P*<0.05 for MAT≥ 100 vs MAT≥ 400.

^5^neutrophil gelatinase-associated lipocalin (NGAL), kidney injury molecule–1 creatinine ratio (KIM–1/Cr), and monocyte chemoattractant protein–1 (MCP–1).

**Fig 4:**
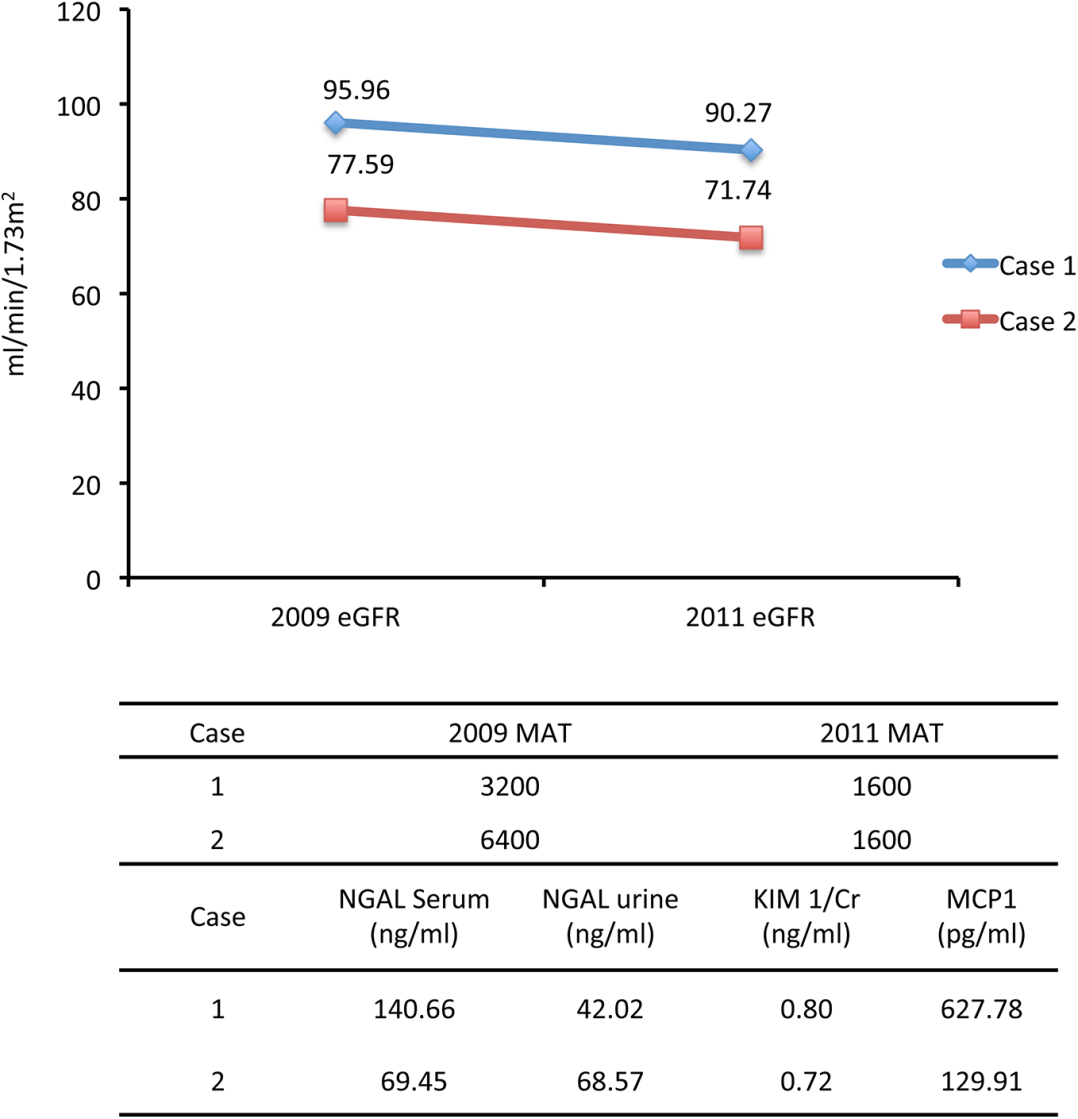
The eGFR, MAT titer and kidney injury marker changes in two cases with leptospira DNA positive urine. (Case I: dark blue; Case 2: light blue).

Among the cohort participants, two individuals (2.3%) presented with asymptomatic persistent high titers of MAT (≥1600) were detected to have positive urine leptospiral DNA indicating a carrier shedding status, and associated deteriorating renal function during two years follow-up (Fig 4).

## Discussion

Fuelled by global warming and climate change, leptospirosis, an important re-emerging infectious disease, imposes challenges not only in tropical or subtropical regions, but also in temperate regions [7,26]. Sporadic reports showed that tubulointerstitial nephritis caused by leptospira might lead to CKD [10,27]. However, the established association between chronic leptospiral infection and CKD is still lacking. This has become a critical question for CKD as a pressing public health burden all over the world. Using a multistage community-based cross-sectional survey and a cohort in the endemic town, this study, to our knowledge, is the first to establish the association between leptospiral infection and chronic kidney disease. In the two-year cohort of the Township, we further found that a higher MAT titer level (MAT titer ≥400) is associated with a higher kidney injury marker KIM–1/Cr, suggesting a possible leptospirosis-associated deterioration of renal function over time.

Chronic kidney disease is associated with hypertension, diabetes mellitus, cardiovascular disease, increased body-mass index, age, and smoking [3]. Additionally, while Chinese herbs containing aristolochic acid can lead to renal damage or even End-Stage Renal Disease, herbal use has been common in Taiwan [28]. In the current study, after adjusting for the abovementioned risk factors, the correlation between CKD and leptospiral infection remained statistically significant. Although participants with a positive anti-leptospira antibody had a significantly higher percentage of diabetes at baseline, the correlation between the renal function and the leptospiral exposure persisted after accounting for the presence of diabetes. As compared between patients with and without diabetes mellitus in multivariate analysis, leptospiral infection still associated with decreased renal function in both groups. The mean age was also higher in the positive group. In Table 2, however, after adjustment with age and the other possible confounders, previous exposure to leptospira is still a significant contributor to the reduction of eGFR. Moreover, we further conducted stratified analyses by age group, the results of which show that previous leptospiral exposure remains significantly associated with a reduced eGFR in the younger (<40 or 40–65 years) but not in the older (>65 years) group (S1 Table). These observations provide consistent evidence that older age per se could not fully explain for the reduction of eGFR among those with prior leptospiral exposure history, particularly in the young and middle-aged groups. Our results indicate that leptospira infection may be associated with, and have likely contributed to prevalent chronic kidney disease at least in some local towns of southern Taiwan.

From the cohort of the endemic Township, 86.4% of participants were seropositive for anti-leptospira antibody at baseline, which is much higher than the 33.9% seropositive rate in the southern county as surveyed in this study. High sero-prevalence rate has been noticed in some sub-tropical regions. A random sample of 1067 persons in Seychelles, Indian Ocean showed a sero-prevalence rate of 37% [29], whereas 54% sero-prevalence rate was observed among healthy population from the Andaman Island, India [30]

The high sero-prevalence rate found in this study might be related to severe flooding during the devastating typhoon Morakot and environmental exposure to leptospira such as the highly concentrated pig farms in this Township area. Asymptomatic infection due to contacting contaminated water or soil after typhoon flooding could also contribute to this observation. People in township receive their water from private ground water wells instead of chlorinated tap water provided in the county, we thus cannot rule out the high prevalence is partially caused by contaminated well water. We also found that participants who maintained a high anti-leptospira antibody MAT titer (≥400) over the two-year follow-up period showed a significant deterioration of renal function, which represent potential risk of CKD in the long run [31]. Since the cohort participants did not report a recognizable history of acute leptospiral infection at the initial enrolment, it is conceivable that asymptomatic leptospiral infection or infection with minor symptoms were most likely the case of these participants after flooding. For kidney involvement, extensive studies suggest that in a progressive nature of kidney that has been damaged, renal function may continue to deteriorate. This is usually contributed by common secondary factors, which are unrelated to the initial disease [32–34]. Therefore, the deterioration of renal function may persist, even after the termination of carrier or chronic infection status. Our focus on *Leptospira santarosai* serovar Shermani as it is the major pathologic serovar identified in both animals and humans in Taiwan. As titers of MAT following acute infection may be extremely high initially and fall to lower levels in months or years, a titer of ≥100 is usually used as evidence of past exposure [4]. Two participants with a persistently high MAT titer excreted leptospira from their urine two years after the presumable primary infection during the flood in 2009, suggesting that a repeatedly high MAT titer level may be due to a failure in clearing the pathogen. Interestingly, these two participants also disclosed a decreased renal function.

A recent report of chronic neuroleptospirosis due to *Leptospira santarosai* was diagnosed using next generation sequencing referring to our previously published genome sequence, suggesting a chronic, persistent infection potentially caused by this serovar.[14,35] Since chronic leptospiral infection may be one of the reasons for CKD, it is worth investigating whether and how leptospiral infection may lead to CKD.

KIM–1, a putative epithelial cell adhesion molecule, expressed in dedifferentiated proximal renal tubular epithelial cells in damaged regions [36,37] was increased in the group with high titers of anti-leptospira antibody. However, the other acute injury markers such NGAL and MCP1 did not show a significant difference. KIM–1 is associated with renal fibrosis and damaging in chronic renal disease besides acute kidney injury [38,39]. The increase of KIM–1/Cr ratio in the group with high MAT titer in 2011, two year after the flood-related outbreak, may reflect and indicate chronic damages including fibrosis process in the kidney (Fig 3). This suggests that chronic injury and fibrosis, a common pathological alteration in progressive CKD, may be the major contributor to CKD after leptospiral infection either via persistence injury or a sequela after acute infection under an indolent process [40]. This conjuncture found support in previous experiments by Tian et al., who showed that the outer membrane protein of *Leptospira santarosai* serovar Shermani can stimulate extracellular matrix production through a TLR2-associated cascade [41,42], revealing possible underlying mechanisms of tubulointerstitial fibrosis after leptospiral infection.

Our study has several strengths. First, the population-based survey design suggests a better generalizability and less selection bias than a hospital-based clinical series. Second, prospective data collection in the two-year cohort study has effectively minimized the likelihood of missing data and misclassification. Moreover, the large sample size of the cross-sectional survey allows us to adjust for several important confounding factors that might also predispose to or worsen CKD while determining the independent association between a previous leptospiral infection and prevalent CKD. Finally, we also conducted a prospective cohort to monitor the renal function change over time after a leptospirosis outbreak to further confirm our observations from the cross-sectional survey. This study was limited in that the case number of the prospective cohort was not large enough to allow for a full adjustment for well-recognized confounders such as age, diabetes mellitus etc. In addition, our study was conducted in a single geographical region endemic with a single serotype- *Leptospira santarosai* serovar Shermani. Whether other serovars exert different effects on the deterioration of renal functions needs to be further confirmed in a prospective manner. We are currently planning for interventional studies, such as antibiotic therapy in a larger cohort in the future.

In a population-based community survey, our findings suggest that previous exposure to leptospira is associated with the higher prevalence and severity of CKD. In an independent two-year cohort, leptospirosis is associated with a continual deterioration of renal function over time, especially in those with a persistent high MAT titer levels. In view of CKD as a global burden, it is worth paying attention to the relationship between CKD and the exposure to leptospira. Our results support a public health policy of screening renal function among at risk residents in leptospira-endemic areas. Also, for effective early prevention, further studies will be needed to clarify the mediating mechanisms and the role of chronic leptospirosis in causing CKD or in its progression. Finally, this study also shed light on the search of underlying factors in areas experiencing CKD of unknown aetiology (CKDu).

## Supporting Information

S1 Checklist STROBE Checklist. (DOC)

S1 Table Multivariate Linear Regression on eGFR Stratified by Age. (DOCX)
