# Supplementary material for: Overlooked Risk for Chronic Kidney Disease after Leptospiral Infection: A Population-Based Survey and Epidemiological Cohort Evidence
Source: PLoS Negl Trop Dis. 2015 Oct 9;9(10):e0004105. doi: 10.1371/journal.pntd.0004105 (PMC4599860; doi:10.1371/journal.pntd.0004105)
Supplement: S1 Table — (DOCX) [file pntd.0004105.s002.docx]

S1 Table. **Multivariate Linear Regression on eGFR Stratified by Age.**

| **Age** | **Number** | **β coefficient of Seropositive to *Leptospira*** | ***P* Value of Seropositive to *Leptospira*** |
| --- | --- | --- | --- |
| <40 | 1021 | -3.43 | 0.001 |
| 40-65 | 1487 | -3.25 | 0.000 |
| >65 | 537 | -1.69 | 0.223 |
